# Impact of closure of educational institutions due to COVID-19 lockdown on overall subjective wellbeing of adolescents and youth: Cross-sectional survey, India

**DOI:** 10.3389/fpsyg.2022.903044

**Published:** 2022-08-12

**Authors:** Tina Rawal, Vijay Kumar Mishra, Shefali Godura Sharda, Kiran Sharma, Rajesh Mehta, Muralidhar M. Kulkarni, Sonu Goel, Monika Arora

**Affiliations:** ^1^Health Promotion Division, Public Health Foundation of India, New Delhi, India; ^2^World Health Organization (WHO), New Delhi, India; ^3^WHO Regional Office for South-East Asia (SEARO), New Delhi, India; ^4^Manipal Academy of Higher Education, Manipal, India; ^5^School of Public Health, Post Graduate Institute of Medical Education and Research, Chandigarh, India

**Keywords:** adolescent, COVID-19, subjective wellbeing, educational institution, India

## Abstract

**Background:**

Students were confined to their homes due to the national closure of educational institutions during the COVID 19 pandemic, thus presenting an unprecedented risk to children’s education, protection, and wellbeing.

**Aim:**

This study aimed to understand the determinants of subjective wellbeing of adolescents and youth (aged 11–21 years) during the COVID-19 pandemic in India.

**Materials and methods:**

A cross-sectional web-based survey was adapted, pre-tested, and finalized to obtain the participant’s responses from schools and colleges. Participants aged 11–17 years were engaged through schools. Consent procedures were followed. The survey link was disseminated through social media for the participants aged 18–21 years. The survey was made available in English and Hindi. The data was collected from March-June, 2021.

**Results:**

Overall, 1,596 students completed the survey. Out of 1,596 students, 1252 (78%) were below 18 years and 344 (21.5%) participants were 18 years and above. Results suggest a statistically significant (*p* < 0.01) difference in the level of student’s life satisfaction before and during the COVID-19 pandemic. Of the students who were dissatisfied with their general life during the pandemic, nearly 63.4% felt sadness followed by other feelings, i.e., boredom (around 60.5%), loneliness (63.7%), and anxiety (62.2%).

**Conclusion:**

This study highlights the need for innovative strategies for adolescents and parents to adopt and promote overall subjective wellbeing, especially during public health crises such as the COVID-19 pandemic.

## Introduction

The COVID-19 pandemic has created unprecedented challenges for people all around the world. In the current scenario, the main measure to contain the COVID-19 infection spread includes self-isolation, quarantine, mask-wearing, hand washing, and social distancing (WHO, 2020). Children of all ages are susceptible to COVID-19 infection, including adolescents (Covid et al., 2020; Dong et al., 2020). In India, children and adolescents have been confined to their homes due to the national closure of educational institutions under lockdown (since mid-March 2020), thus presenting an unprecedented risk to children’s education, protection, and wellbeing. These closures have also exposed adolescents and youth to several risk factors including unhealthy living habits and mental health issues (Lee, 2020).

In India, the new academic year (2020–2021) was initiated virtually in April 2020 in New Delhi and in June 2020 in other parts of the country. While this shift to virtual learning has been able to provide some semblance of normalcy for students, who are otherwise holed up in the safety of their homes, it has also exposed widespread social and economic disparities in society. This is reflected by the inequitable distribution and capacity to access digital platforms due to the family’s absence of multiple electronic devices and limited financial capacity to afford electronic devices (Ray et al., 2022). Further impediments are the limited skill set among parents to navigate digital platforms, appropriately trained school staff, and disruption in power supply, internet, and Wi-Fi connections (Nesar et al., 2021). In this scenario, the already unfavorable conditions for adolescents and youth are further aggravated, leading to mental stress, trauma, and, in several cases, social and emotional stigmatization as is evident in some global literature (Shah et al., 2020; Berasategi Santxo et al., 2021). Even among students who have access to and are adept at using digital devices and platforms, there is differential acceptance of e-learning assimilation and learning methodologies (Ray et al., 2022), both leading to sub-optimal and compromised teaching-learning processes. Factors influencing learning include physiological factors, psychological factors, environmental factors, and methodology of instructions. Findings of a study conducted by the Indian Psychiatry Society suggests that more than two-fifths of people were experiencing common mental disorders due to the COVID-19 pandemic (Grover et al., 2020). It has remarkably affected the lives of adolescents and has impacted their mental health conditions due to online classes, less play time, more screen time, and limited interactions with peers. Evidence from developed countries has revealed that home confinement resulted in increased screen time which is associated with sedentary behavior, snacking, and poor sleep patterns (Ten Velde et al., 2021) which may result in anxiety, exhaustion, emotional disturbance, and stress. The result of another study conducted to analyze the wellbeing of adolescents in Spain revealed intermediate scores in terms of physical activity and emotional and academic domains (Berasategi Santxo et al., 2021).

The primary objective of this study is to explore the determinants of subjective wellbeing of adolescents (11–17 years) and youth (18–21 years) during the COVID-19 pandemic. The secondary objective is to understand the changes in the subjective wellbeing of adolescents before and during the pandemic.

## Materials and methods

### Study design

A cross-sectional survey was conducted using an online platform to understand the general life satisfaction of school/college students before and during the COVID-19 lockdown across 15 Indian states. The survey responses were self-reported. The survey used in one of the studies conducted by the University of Luxemburg (de Abreu et al., 2021) was adopted in the Indian context to study the effects of prolonged social isolation coupled with the challenges of home-schooling/online classes on adolescents and youth’s subjective wellbeing and resilience skills. The adapted survey was reviewed by experts at PHFI, WHO SEARO, and WHO country office for India and pre-tested with participants from schools and colleges. The survey was made available in English and Hindi.

### Participants

The study included participants from schools and colleges in the age group of 11–21 years. The participants under study belonged to the selected schools across 15 states/union territories (Bihar, Chandigarh, Chennai, New Delhi, Haryana, Himachal Pradesh, Karnataka, Kerala, Madhya Pradesh, Maharashtra, Manipur, Punjab, Rajasthan, Uttar Pradesh, and Uttarakhand) of India. Overall, 1,596 students completed the survey.

### Data collection

The data was collected through web-based forms, which were circulated through social media, such as Facebook and WhatsApp, for the participants aged 18–21 years. The online survey form included nearly 83 questions including socio-demographic characteristics of the respondents. The survey link was open for participants aged 18 and above. Participants aged 11–17 years were engaged through schools. Consent procedures were followed. The survey link was shared with the students by the school authority following school consent. Informed assent was obtained from the participants below 18 years and consent was obtained from participants above 18 years. The study was approved by PHFI IEC (TRC-IEC 446.1/20). The data was collected from March–June, 2021.

### Measures

The explanatory variables used in this study were: age, gender, father/mother’s education, father/mother’s occupation, wealth index, physical activity, and psycho-social feelings (sad/worried/bored/lonely) during COVID-19. The wealth of the adolescent’s households was assessed by asking them questions about various material assets (television, car, electricity, moped/bicycle, built-in kitchen sink, hot running water, washing machine, refrigerator, mobile/cellular phone, separate room for study, computer, and internet access).

The wealth index (a composite measure of a household’s cumulative living standard) was created based on the suggested guideline of DHS (demographic and health survey). We have considered responses on households’ material possessions (car, tractor, radio, chair, TV, table, refrigerator, etc.) to create a wealth index using principal component analysis (PCA), scoring then converting into “5 quintiles,” and further (as suggested by DHS).

We used 14 items-each representing an individual response (yes = 1, no = 0) on a distinct household item. The questions to assess the household’s assets were: Does your household have-electricity/a fixed telephone/television/radio/refrigerator/car/moped/scooter/motorcycle/washing machine/Internet/computer/smart phone/tablet.

The PCA was used to generate scores using responses on household assets. The score was further converted into 5 quintiles using the function “xtile” in Stata software. In this way, we created the variable “wion” with five quintiles. These five categories were labeled as poorest = 1, poorer = 2, 3 = medium, 4 = richer, and 5 = richest, as per the guidelines suggested by the DHS on the creation of wealth index/wealth quintile.

The responses (dichotomous) on these households’ assets were used to create the variable “wealth index” using PCA. The scores generated through PCA were converted into 5 quintiles. The 5 quintiles were then further divided into three categories: poor, medium, and rich. The variable “wealth index” was used to understand the socio-economic status (SES) of adolescents.

### Dependent variable

The dependent variable in this study was “general life satisfaction during the pandemic,” which was assessed by the question “How satisfied are you generally with your life during the time of the coronavirus?” This question included four response categories, i.e., very dissatisfied, not satisfied, satisfied, and very satisfied. We created the variable “gen_life_satis” by dichotomizing the variable as 0 = satisfied (satisfied/very satisfied) or 1 = dissatisfied (very dissatisfied/not satisfied) for analysis purposes.

### Independent variable

The variables “father_occupation,” “wealth_index,” “Psysc,” and “Phy_active” were considered the main outcome variables in the logistic regression modeling. The variable “father_occupation” was assessed by the question What is your father’s occupation?

The question had seven closed-ended options: Professional (e.g., doctors, lawyers, engineers, etc.); Semi-Professional (technicians, assistants, etc.); clerical, shop owner, or farmer; Skilled worker (with formal training or certificate); Semi-skilled worker (without any formal training or certificate); Unskilled worker (Laborer); and Unemployed. We have recategorized this variable into four categories: 1 = professional (professional/semi-professional), 2 = skilled (skilled/semi-skilled worker), 3 = unemployed, and 4 = other (clerical, shop owner, farmer/unskilled worker). Similarly, the variable “wealth_index” was created by recategorizing the variable “wion” into 1 = poor (poorest/poorer), 2-medium, and 3-rich (richer/richest). The independent variable “Psysc” was created through the responses of four items (sad/worried/bored/lonely) which were dichotomized (0 = no, 1 = yes) for analysis purposes. We also created a variable named “Phy_active” by simply using the dichotomous response to the question Did you perform PT/drill during COVID-19 lock-down?

### Data analysis

Descriptive analyses including exploratory graphs and percentage distribution of categorical variables were done to understand the distribution of data. Bivariate analysis using non-parametric tests, i.e., Chi-square at a 5% level of significance, was used to understand the association between outcome and predictors. The absolute changes were calculated to understand the changes in the wellbeing of adolescents before and during the pandemic. The negative effects (sad/worried/bored/lonely) during COVID-19 were assessed with a four-item scale, adapted from the International Survey of Children’s Well-Being. Adolescents were asked to rate the frequency with which they experienced specific feelings along a 4-point scale ranging from “almost never (0)” to “very often (Covid et al., 2020).” The responses to the four items (sad/worried/bored/lonely) were dichotomized (0 = no, 1 = yes) for analysis purposes. Bivariate logistic regression models were used to study the strengths of association between predictors (occupation of father, wealth index, psych, and physical activity) and the dichotomous outcome variable (general life satisfaction).

The variables “age” and “gender” were treated as the co-variates in the predictive modeling. We also estimated the predicted score, margin, and interaction effect of covariates, which was illustrated through a “marginal plot.”

## Results

Out of 1,596 students, 1252 (78%) were below 18 years and 344 (21.5%) participants were 18 years and above. Around 61.4% of female and 38.6% of male participants responded to the survey, as shown in Table 1. More than half of the parents (mother 51% and father 56.5%) of the respondents were graduates or had advanced degree qualifications. Our study found that most of the students’ mothers were homemakers or housewives (66.8%), while fathers were skilled (42.5%) or professional workers (38.9%) in different organizations/multi-national companies across 15 Indian states. More than one-third of students (40.3%) belonged to the poor socioeconomic status of the society, while an almost equivalent proportion of the students (39.7%) belonged to richer households based on wealth quintiles. The proportion of the respondents belonging to the middle status of the society was relatively lesser (about 20%) compared to poor and rich. The mean age of the respondents in this study was 15.4 years (Table 1).

**TABLE 1:** Socio-demographic profile of the respondents (*n* = 1596).

Socio-demographic profile indicators	Mean (SD)/*n* (%)	95% CI	
Age	15.42 (2.88)	15.28	15.56
**Age group**			
<18 years	1252 (78.45)	76.36	80.40
≥ 18 years	344 (21.55)	19.60	23.64
**Gender**			
Male	616 (38.60)	36.23	41.01
Female	980 (61.40)	58.99	63.77
**Highest education attained by mother**			
Advanced/professional degree	263 (16.48)	14.74	18.38
Graduate	551 (34.52)	32.23	36.89
≤ HSC (primary/secondary/higher secondary)	782 (49.00)	46.55	51.45
**Highest education attained by father**			
Advanced/professional degree	304 (19.05)	17.19	21.05
Graduate	598 (37.47)	35.12	39.87
≤ HSC (primary/secondary/higher secondary)	694 (43.48)	41.07	45.93
**Occupation of mother**			
Professional	264 (16.54)	14.80	18.45
Skilled	214 (13.41)	11.82	15.17
Homemaker (housewife)	1066 (66.79)	64.44	69.06
Other	52 (3.26)	2.49	4.25
**Occupation of father**			
Professional	622 (38.98)	36.61	41.39
Skilled	679 (42.54)	40.14	44.99
Unemployed	212 (13.28)	11.70	15.04
Other	83 (5.20)	4.21	6.41
**Wealth index**			
Poor	644 (40.35)	37.97	42.78
Middle	319 (19.99)	18.09	22.02
Rich	633 (39.66)	37.28	42.08
Total	**1596 (100)**		

**FIGURE 1:**
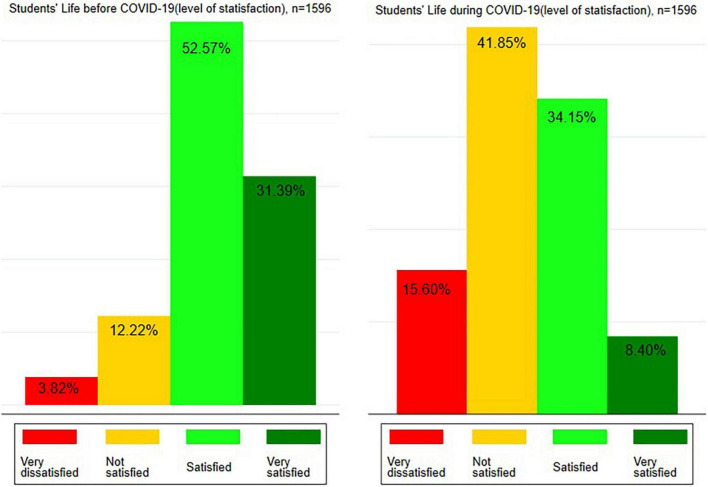
Students’ life before and during COVID-19 (level of satisfaction).

More than three quarters of the students (84.0%) were satisfied/very satisfied with their general school life before the COVID-19 pandemic. This study found that the proportion of dissatisfaction among students increased during COVID-19. More than half of the students (57.5%) felt dissatisfied with their life at home during lockdown (Figure 1). The result of the non-parametric test (Wilcoxon signed-rank) on two related samples suggests that there is a statistically significant (*p* = 0.000) difference in the level of student’s life satisfaction before and during the COVID-19 pandemic. The value of absolute change reveals that the level of student’s life satisfaction has decreased due to the pandemic, which resulted in increasing dissatisfaction in general lifestyle, including a longer stay at home and online schooling during COVID-19 (Supplementary Table 1).

**FIGURE 2:**
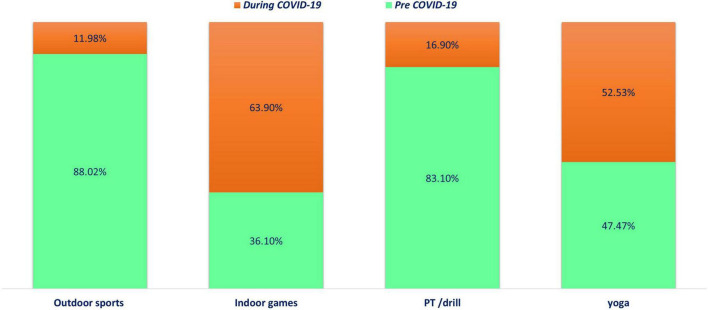
Activities performed in physical education/PT period in school pre and during COVID-19.

Most of the students played (88%) outdoor sports (football, cricket, etc.) and did PT/drill (83.1%) before COVID-19, while relatively fewer students played outdoor sports (12%) due to lockdown caused by COVID-19 in their respective areas. This study found that the lockdown has changed the regular playing habits of the students. The proportion of students playing indoor games has increased from 36.1% (before COVID-19) to 63.9% during COVID-19 restrictions (Figure 2). Binomial tests were applied to test whether the proportions of students playing games (outdoor sports, indoor games, PT/drill, and yoga) differ significantly before and during the COVID-19 pandemic. Tests suggest that there are statistically significant differences between proportions of students playing outdoor sports (*p* = 0.000), indoor games (*p* = 0.000), and PT/drill (*p* = 0.000) for before and during COVID pandemic lockdown. While no statistically significant difference was found for yoga (*p* = 0.210) before and during the COVID period (Supplementary Table 2).

This study found that the overall proportion of students who were dissatisfied with their general lives during the pandemic increased from 16.0% (before COVID-19) to 57.5% (during COVID-19).

The respondents who felt dissatisfied during the pandemic who were younger than 18 years (61.4%) were more than those over 18 years (43.0%). While the proportion of dissatisfied female students with their general lives during the pandemic was about 60%, which was relatively higher than their male counterparts (53.2%). During the pandemic, more than half of the students with unemployed fathers felt dissatisfied with their general lives at home. The proportion of students who belonged to the middle (53%) and richer (56%) sections of the society was relatively lesser than those who had a poor SES (61.5%). This study found that the education of parents was not significantly associated with the level of dissatisfaction among students during the pandemic, while other variables, such as age group (*p* = 0.000), gender (0.007), occupation (0.002), and wealth index (*p* = 0.020), were significantly associated with the outcome variable (general life satisfaction). Also, this study found a significant increase in dissatisfaction levels among students during the COVID-19 lockdown in their respective areas (Table 2).

**TABLE 2:** Percentage of respondents who were dissatisfied with their general life, before and during COVID-19 with Chi-Square test of association.

Background characteristics	Before COVID-19	95% CI		During COVID-19	95% CI		Chi-sq.(*P*-value)
	dissatisfied			dissatisfied			
**Age group**	*n* = 256			*n* = 917			
<18 years	15.65	13.74	17.78	61.42	58.69	64.08	*p* = 0.000
=>18 years	17.44	13.78	21.83	43.02	37.88	48.33	
**Gender**						
Male	16.07	13.37	19.19	53.25	49.29	57.16	*p* = 0.007
Female	16.02	13.85	18.46	60.10	57.00	63.13	
**Highest education of mother**					
Advanced/professional degree	16.73	12.68	21.75	53.61	47.55	59.57	*p* = 0.353
Graduate	13.25	10.66	16.35	57.53	53.35	61.60	
≤ HSC (primary/secondary/higher secondary)	17.77	15.25	20.62	58.70	55.20	62.10	
**Highest education of father**					
Advanced/professional	12.17	8.94	16.36	52.96	47.32	58.52	*p* = 0.201
Graduate	16.56	13.78	19.76	58.03	54.02	61.93	
≤ HSC (primary/secondary/higher secondary)	17.29	14.65	20.29	58.93	55.23	62.54	
**Occupation of mother**		
Professional	14.02	10.32	18.76	50.76	44.73	56.76	p = 0.055
Skilled	19.63	14.83	25.51	59.81	53.09	66.19	
Homemaker/housewife	15.67	13.60	17.98	58.16	55.17	61.09	
Other	19.23	10.60	32.34	67.31	53.41	78.71	
**Occupation of father**						
Professional	14.47	11.91	17.46	52.57	48.63	56.48	*p* = 0.002
Skilled	15.91	13.34	18.86	58.32	54.57	61.98	
Unemployed	20.28	15.39	26.25	66.04	59.38	72.11	
Other	18.07	11.16	27.92	65.06	54.17	74.57	
**Wealth_index**						
Poor	18.79	15.95	22.00	61.49	57.66	65.18	*p* = 0.020
Middle	10.97	7.98	14.91	52.98	47.47	58.41	
Rich	15.80	13.16	18.86	55.61	51.71	59.44	
Total (*N* = 1596)	**256 (16.04)**	**14.3**	**17.93**	**917 (57.46)**	**55.01**	**59.86**	

p < 0.05 indicates statistically significant difference, AC, absolute change, “ + ve” sign indicates increasing dissatisfaction due to pandemic, AC (%) = % (during COVID-19)-%(before COVID-19).

The heat map-based cross-tab visualization illustrated that more than half of the students felt various psycho-social negative feelings during the COVID-19 lockdown across selected Indian states. Of the students who were dissatisfied with their general life during the pandemic, nearly 63.4% felt sadness followed by other feelings, i.e., boredom (around 60.5%), loneliness (63.7%), and anxiety (62.2%) (Supplementary Figure 1).

The results of the logistic regression revealed that the students with unemployed fathers [AOR-1.73, 95% CI (1.25–2.40)] were significantly more likely to feel dissatisfied with their general lives during the pandemic, compared to the students whose fathers were professional workers. The odds of dissatisfaction were lower among the students who belonged to middle socioeconomic status [AOR-0.70, 95% CI (0.53–0.92)] or richer compared to those who belonged to poor sections of the society. Students who felt sad/bored/lonely/anxious were 2 times more [AOR-2.14, 95% CI (1.40–3.28)] likely to be dissatisfied with their general lives compared to those who did not feel such negative feelings. The students who did PT/drill were less likely to feel dissatisfied than those who did not do any physical activity during the pandemic (Table 3).

**TABLE 3:** Results of Logistic regression showing determinants of general life satisfaction among students during the pandemic.

General life satisfaction (dependent)	AOR	Std. Error	*p*-value	(95% Conf. Interval)	
				LL	UL
**Father’s occupation**					
Professional^®^	1.00				
Skilled	1.28	0.14	0.029	1.03	1.60
Unemployed	1.73	0.29	0.001	1.25	2.40
Other	1.59	0.39	0.060	0.98	2.56
**Wealth_index**					
Poor^®^	1.00				
Medium	0.70	0.10	0.011	0.53	0.92
Rich	0.83	0.10	0.099	0.66	1.04
**Psysc (sad/bored/lonely/anxious)**					
No^®^	1.00				
Yes	2.14	0.47	0.000	1.40	3.28
**Phy_active (pt_drill)**					
No^®^	1.00				
Yes	0.92	0.19	0.679	0.62	1.37

^®^: Reference category, AOR, adjusted odds ratio, 95% CI, LL-lower limit, UL-upper limit.

The age and sex interaction modeling and margin plot provided us with the probability of dissatisfaction among students by age and gender. On average, the probability of dissatisfaction among male students below 18 years is 0.58 (58%), while it is 0.62 (62%) among female students in the same age group (Supplementary Table 3).

## Discussion

The findings of our study revealed that there is a statistically significant (*p* = 0.01) difference in the level of student’s life satisfaction before and during the COVID-19 pandemic. There are statistically significant differences between proportions of students playing outdoor sports (*p* = 0.000), indoor games (*p* = 0.000), and PT/drill (*p* = 0.000) before and during the COVID pandemic lockdown. Of the students who were dissatisfied with their general life during the pandemic, nearly 63.4% felt sadness followed by other feelings, i.e., boredom (around 60.5%), loneliness (63.7%), and anxiety (62.2%). Students who felt sad/bored/lonely/anxious were 2 times more [AOR-2.14, 95% CI (1.40–3.28)] likely to be dissatisfied with their general lives compared to those who did not feel such negative feelings.

Some of the major indicators of wellbeing in the adolescent age bracket include health-related quality of life, teachers’ opinion of academic performance, and self-perceived health (Guevara et al., 2021). The COVID-19 lockdown imposed sudden and unprecedented restrictions on communities and people worldwide, which disrupted the daily routines of every individual and thus created economic, social, educational, and health, including psychological, consequences in general. The adolescents and youth also faced these challenges (Shah et al., 2020). This study was hence carried out to understand such emerging issues by finding the determinants of *subjective wellbeing* of adolescents and youth during COVID-19 and also to understand the *changes in their subjective wellbeing* before and during the pandemic. The study was able to delineate that the (i) variables like age group, gender, occupation, and wealth index were the determinants of general life satisfaction, (ii) students with unemployed fathers and students from poor economic strata of the society were dissatisfied more, (iii) the level of dissatisfaction among female students below 18 years was more compared with the male students in the same age group, and (iv) students who did some sort of physical activity felt less dissatisfaction in general. A similar study was conducted to investigate the correlates of different dimensions of subjective wellbeing in adolescents (10–16 years) from Luxembourg, Germany, and Brazil, which revealed that adolescents from low-income homes may be more vulnerable to the negative impacts of COVID-19 that can affect mental health (de Abreu et al., 2021).

The economic consequences and financial constraints due to the pandemic and the lockdown impacted some families. We found in our study that students with unemployed fathers and students from poor economic strata of the society were dissatisfied more. Similar findings were reported (Fegert et al., 2020) in a study that significant stressors such as unemployment, income decline, and unmanageable debts typically harm the wellbeing of parents, influencing parent-child relationships and increasing children’s risk of mental health problems. Their study explained that there are several indicators that socioeconomically disadvantaged children and adolescents are at the highest risk for COVID-19 associated mental health effects. It was explained that disadvantageous circumstances in one context often amplify adverse conditions in other contexts. The result of another study conducted with adolescents revealed that heavy restrictions placed on activities that promote wellbeing and concerns about infection may result in a reduction in life satisfaction and subjective wellbeing (Soest et al., 2020). Findings of a study conducted with Spanish families (who had one or more children aged 0–12 years) revealed that factors like conditions of the house and place of residence also influence educational activities such as reading, physical activity, free play, or the use of technological devices between children, e.g., those residing in urban and rural settings or in houses with a garden or in small or large flats (Zagalaz-Sánchez et al., 2021).

We also found that the level of dissatisfaction among female students below 18 years was more compared with the male students in the same age group. Findings of a study (Zhou et al., 2020) also found that the female gender was at a higher risk factor for depressive and anxiety symptoms. Another study also depicted similar results (Magson et al., 2021). They found that adolescents experienced significant increases in depressive symptoms and anxiety, and a significant decrease in life satisfaction, which was particularly pronounced among girls.

In our study, students who did some sort of physical activity felt less dissatisfaction in general. Similar findings were reported in other studies (Brand et al., 2020; Martarelli and Wolff, 2020) and indicated that those who were inactive during a lockdown had worse subjective wellbeing compared to others. This is an important result as low mood states are linked with less self-control, which in itself is shown to be an important determinant of following restrictive rules such as social distancing. Therefore, policymakers can use such outcomes to promote physical activity and exercises in their countries to derive gains from its positive effects on mood under similar lockdown restrictions in the future. The result of a study highlighted (Stubbe et al., 2007) that exercise participation is associated with higher levels of life satisfaction and happiness which is again in line with our study findings.

### Strengths and limitations

The main advantage of this study is the usage of a rich cross-sectional dataset based on a previous national survey. It provided us a chance to explore potential psychological effects across socioeconomic groups of the study population. The subjective wellbeing and adolescents’ negative emotions were measured in a standardized manner by using validated tools.

This investigation has some limitations. The first involves the cross-sectional nature of the study, thus it is not possible to infer causal relationships. Secondly, the sample was not randomly selected. The online survey captured the responses based on recall questions, instead of longitudinally tracking the affective state of adolescents and youth. Also, it is very difficult to determine the presence or the magnitude of recall bias in self-reported subjective wellbeing indicators. It has been mentioned that people over/underestimate their past emotions. Another limitation is that our sample was composed of adolescents and youths who had online access, which was used given the circumstances and constraints of the lockdown. As the study was conducted considering time-sensitivity, other validated instruments were not available to refer to. Also, there may be a gender bias as females are the majority in the sample.

### Implications for policy and practice

Adolescents’ and young people’s lives were disrupted due to the COVID-19 pandemic, impacting their social, emotional, and economic wellbeing. Its negative outcomes have unfolded rapidly and arbitrarily in the areas of education, employment, physical and mental health, and general wellbeing. This study was conducted to generate evidence and support the stakeholders to manage the possible short- and long-term consequences of school closure and social isolation on young people in India following the COVID-19 outbreak. The study highlighted the need to strengthen the teaching approaches by introducing or modifying the existing training courses and syllabus in the rapidly changing environment to make the learning process more effective and meaningful. Also, it suggests the need for provision of flexibility in the school curriculum to lessen the burden on students and parents and increase interactivities during online classes and opportunities to access digital equipment, such as android phones, to economically weaker sections to facilitate the continuous learning process.

## Conclusion

Adolescents are vulnerable and require careful consideration by their caregivers and healthcare system to allow for overall wellbeing, including mental health support, during the lockdown. The current study suggests declining subjective wellbeing among Indian adolescents and youths when compared to pre-pandemic lockdown levels. Students with unemployed fathers and of a poor economic strata in society were more dissatisfied. The level of dissatisfaction among female students below 18 years was more compared with the male students in the same age group and students who did some sort of physical activity felt less dissatisfaction in general. Thus, it is imperative to assess the implications of policies enacted to lessen the impact of the pandemic on the mental health of adolescents and to estimate the risk/benefit ratio of measures, such as homeschooling (online schooling), to be better prepared for future developments (Nagata et al., 2020). The current literature suggests that the pandemic and the lockdown will hit disadvantaged groups of the population the most. Hence, there is a need for social and health policy, public health, and further research focus on such groups. These findings also contribute to basic research in psychology and hence may be of interest to behavioral researchers and interventionists as well.

## Data availability statement

The raw data supporting the conclusions of this article will be made available by the authors on request.

## Ethics statement

The studies involving human participants were reviewed and approved by Public Health Foundation of India-Institutional Ethics Committee (PHFI-IEC) (TRC-IEC 446.1/20). Written informed consent for participation was not provided by the participants’ legal guardians/next of kin because: Adolescents and youth (aged 11–21 years) were invited to participate and complete the web-based online survey during COVID-19 pandemic. Informed assent was completed by all the participants below 18 years of age and informed consent was obtained from participants aged 18 and above. Adolescents below 18 years of age were invited through schools after obtaining consent from school authorities.

## Author contributions

MA, RM, KS, and TR conceptualized the study design and provided guidance to implement the study. TR collected, organized, and wrote the manuscript. VM supported the analysis of the data with TR under the guidance of MA, RM, and KS. MA, RM, KS, SS, MK, and SG revised the manuscript critically for intellectual contents. All authors approved the final manuscript.
