# Supplementary material for: Impact of closure of educational institutions due to COVID-19 lockdown on overall subjective wellbeing of adolescents and youth: Cross-sectional survey, India
Source: Front Psychol. 2022 Aug 12;13:903044. doi: 10.3389/fpsyg.2022.903044 (PMC9415378; doi:10.3389/fpsyg.2022.903044)
Supplement: Supplementary file 1 [file Table_1.DOCX]

Supplementary Material

**Table.1S Before and during COVID-19 (level of satisfaction)**

| Level of satisfaction | Pre COVID-19 | During COVID-19 | AC (%) | p-value |
| --- | --- | --- | --- | --- |
|  | **n (%)** | **n (%)** |  |  |
| **Very dissatisfied** | 61(3.82) | 249(15.60) | 11.78 | p=0.000 |
| **Not satisfied** | 195(12.22) | 668(41.85) | 29.63 |  |
| **Satisfied** | 839(52.57) | 545(34.15) | -18.42 |  |
| **Very satisfied** | 501(31.39) | 134(8.40) | -22.99 |  |
| Total | **1596** | **1596** |  |  |

**Note: p<0.05 indicates statistically significant difference, AC-Absolute change, ‘-ve’ sign indicates decreasing satisfaction**

**Table. 2S: Activities performed in physical education/PT period in school pre and during COVID-19**

| Activity in physical education/PT period | Pre COVID-19(%) | During COVID-19(%) | AC (%) | p-value (Binomial test) |
| --- | --- | --- | --- | --- |
| **Outdoor sports** | 88.02 | 11.98 | -76.04 | p=0.000 |
| **Indoor games** | 36.10 | 63.90 | 27.80 | p=0.000 |
| **PT /drill** | 83.10 | 16.90 | -66.20 | p=0.000 |
| **yoga** | 47.47 | 52.53 | 5.06 | p=0.210 |

**Note: p<0.05 indicates statistically significant difference, AC-Absolute change**

**Table.3S Marginal effect table**

| **general life satisfaction(dependent)** | **Margin** | **Delta-method** | **z** | **P>z** | **[95% Conf.Interval]** | |
| --- | --- | --- | --- | --- | --- | --- |
|  |  | **Std. Err.** |  |  |  |  |
| **gender#agegrp** |  |  |  |  |  |  |
| **male#below18yrs** | 0.58 | 0.02 | 24.66 | 0.000 | 0.54 | 0.63 |
| **male#18yrsandAbove** | 0.44 | 0.04 | 11.76 | 0.000 | 0.37 | 0.52 |
| **female#below18yrs** | 0.62 | 0.02 | 36.22 | 0.000 | 0.59 | 0.65 |
| **female#18yrsandAbove** | 0.47 | 0.04 | 11.8 | 0.000 | 0.39 | 0.55 |
