# Supplementary figures and images for: Impact of closure of educational institutions due to COVID-19 lockdown on overall subjective wellbeing of adolescents and youth: Cross-sectional survey, India
Source: Front Psychol. 2022 Aug 12;13:903044. doi: 10.3389/fpsyg.2022.903044 (PMC9415378; doi:10.3389/fpsyg.2022.903044)

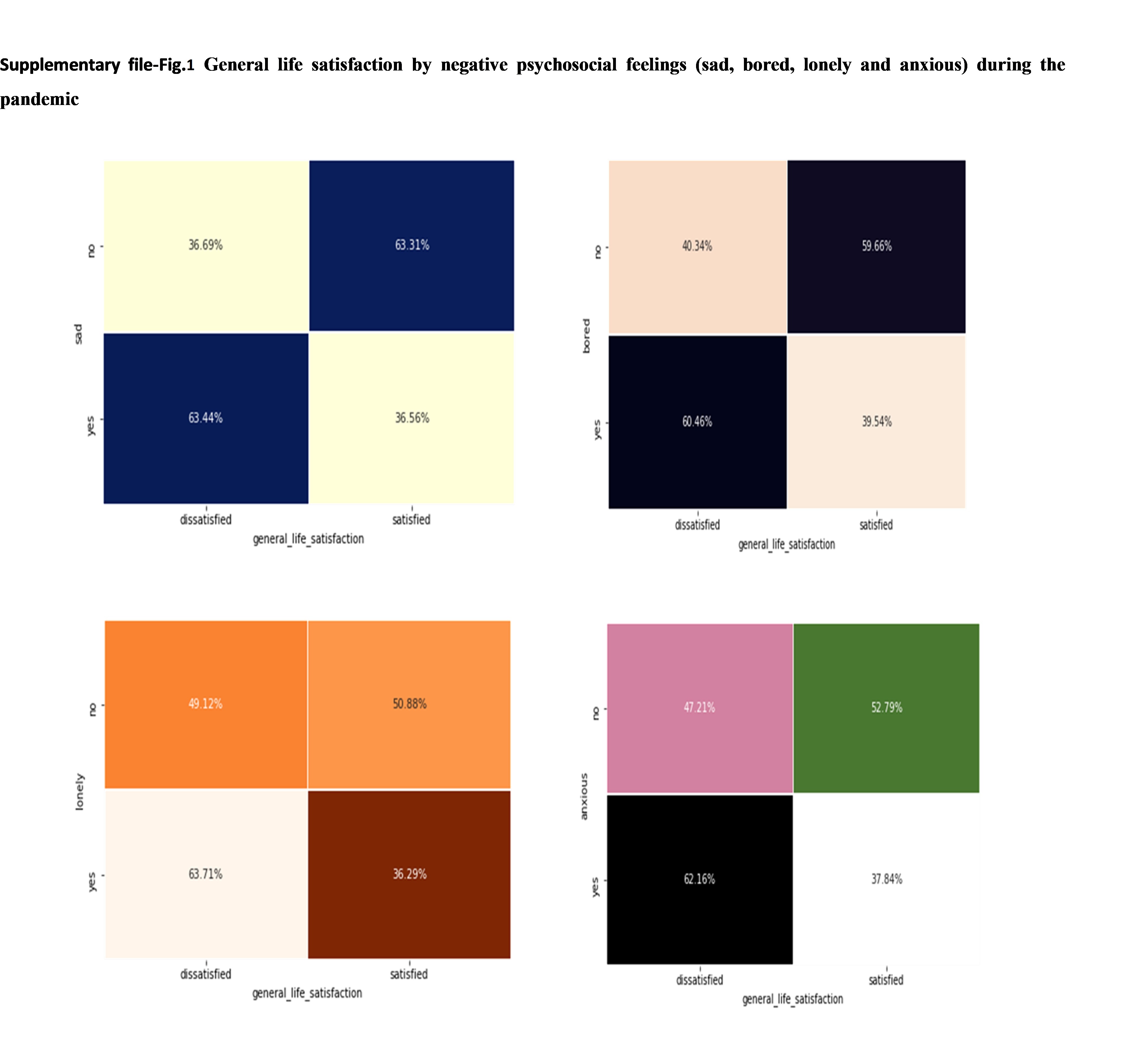

Supplement: Supplementary file 2 [file Image_1.jpg]
